# Serum FGL2 is correlated with the severity and psychological status of diarrhea-predominant irritable bowel syndrome

**DOI:** 10.3389/fmed.2026.1796532

**Published:** 2026-04-28

**Authors:** Fubing Wang, Xin Li, Peipei Zhao

**Affiliations:** 1 Department of Gastroenterology, Nantong Second People’s Hospital, Nantong, Jiangsu, China; 2 Department of Geriatrics, Nantong Second Peoples Hospital, Nantong, Jiangsu, China

**Keywords:** disease severity, fibrinogen-like protein 2, inflammatory cytokines, irritable bowel syndrome, psychological comorbidity

## Abstract

**Background:**

Fibrinogen-like protein 2 (FGL2) is involved in immune-mediated inflammatory responses. However, its role in diarrhea-predominant irritable bowel syndrome (IBS-D) remains to be elucidated. This study investigated serum FGL2 levels in patients with IBS-D and examined their association with inflammatory cytokines, disease severity, and psychological status. Patients with inflammatory bowel disease (IBD), including Crohn’s disease and ulcerative colitis, were excluded to avoid diagnostic overlaps. IBS-D diagnosis was based on the Rome III criteria.

**Methods:**

A total of 117 patients with IBS-D and 110 matched healthy controls were enrolled. Serum FGL2 and inflammatory cytokine levels (TNF-α, IL-1β, IL-6, and IL-17A) were quantified using ELISA. Symptom severity (IBS-SSS), depressive symptoms (PHQ-9), anxiety/depression (HADS), and disease-specific quality of life (IBS-QOL) were assessed. In parallel, publicly available intestinal transcriptome datasets (GEO) were reanalyzed. Statistical analyses included Student’s *t-*test and Pearson’s correlation.

**Results:**

Compared with healthy controls, patients with IBS-D exhibited significantly elevated serum FGL2, WBC, neutrophil, and pro-inflammatory cytokine levels (all *P* < 0.05). Serum FGL2 levels were markedly higher in patients with IBS-D and demonstrated a strong discriminative ability (AUC = 0.935; cutoff = 105.5 ng/mL, sensitivity 82.9%, specificity 91.8%). FGL2 levels were positively correlated with TNF-α (*r* = 0.287), IL-1β (*r* = 0.338), IL-6 (*r* = 0.251), and IL-17A (*r* = 0.213). Higher FGL2 concentrations were also associated with increased symptom severity (IBS-SSS, *r* = 0.241), greater depressive symptoms (PHQ-9, *r* = 0.279), higher HADS scores (*r* = 0.222), and poorer quality of life (IBS-QOL, *r* = 0.198) (all *P* < 0.05). Transcriptomic reanalysis revealed dysregulation of FGL2 expression in IBS intestinal tissues, suggesting altered local immune regulation and a potential shift in the FGL2 isoform balance.

**Conclusion:**

This study provides the first evidence that serum FGL2 levels are elevated in IBS-D and are associated with inflammation, symptom severity, and psychological distress. While the correlations with immune and psychosocial measures are consistent with previous IBS research, the detection of FGL2 and the proposed isoform imbalance hypothesis represent novel findings. These results are exploratory, and causality cannot be inferred. Further validation and mechanistic studies are required.

## Graphical Abstract

**Figure:**
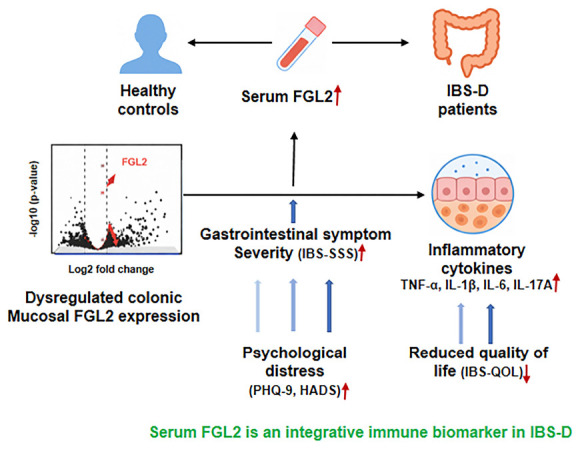
Patients with IBS-D exhibit elevated levels of circulating fibrinogen-like protein 2 (FGL2) compared to healthy controls. Increased serum FGL2 levels were positively associated with systemic low-grade inflammation, as reflected by higher concentrations of pro-inflammatory cytokines (TNF-α, IL-1β, IL-6, and IL-17A). Elevated FGL2 levels were further correlated with greater gastrointestinal symptom severity (IBS-SSS), increased psychological distress (PHQ-9 and HADS), and reduced disease-specific quality of life (IBS-QOL). Reanalysis of publicly available colonic mucosal transcriptomic data demonstrated dysregulated FGL2 expression in intestinal tissues, as visualized by a volcano plot, suggesting altered local immune regulation.

## Introduction

Irritable bowel syndrome (IBS) is a disorder of gut-brain interaction (DGBI) characterized by chronic abdominal pain and altered bowel habits, affecting approximately 10–15% of the global population (1). Among its clinical subtypes, diarrhea-predominant IBS (IBS-D) is associated with a greater symptom burden, impaired quality of life, and a higher prevalence of anxiety and depression than other IBS subtypes (2, 3). Despite its substantial clinical and socioeconomic impact, the pathophysiological mechanisms underlying IBS-D remain incompletely understood, and reliable objective biomarkers for diagnosis, disease stratification, and monitoring are lacking.

Over the past decade, numerous candidate biomarkers have been investigated in IBS, reflecting its multifactorial pathophysiology. These include inflammatory mediators (e.g., cytokines and chemokines), markers of intestinal permeability (e.g., zonulin and intestinal fatty acid-binding protein), neuroendocrine factors, microbe-derived metabolites, and immune cell-related proteins (4–7). Recent systematic reviews and meta-analyses have demonstrated modest but relatively consistent alterations in inflammatory markers, particularly pro-inflammatory cytokines such as tumor necrosis factor-alpha (TNF-α), interleukin (IL)-1β, IL-6, and IL-17A, in subsets of patients with IBS (5, 6, 8). Importantly, immune dysregulation and low-grade inflammation appear more pronounced in IBS-D, with evidence of increased mucosal immune cell infiltration, mast cell activation, and altered innate immune signaling, compared with other IBS subtypes (6, 9, 10). However, these analyses also underscore substantial heterogeneity across study cohorts, variability in diagnostic criteria and sampling strategies, and limited reproducibility of individual biomarkers, highlighting the absence of a single biomarker with sufficient sensitivity or specificity for routine clinical use (4, 7, 8). Consequently, there is growing interest in identifying upstream immunoregulatory molecules that may integrate inflammatory responses, epithelial barrier dysfunction, and neuroimmune interactions, thereby offering improved biological plausibility and translational relevance to IBS (9–11).

Fibrinogen-like protein 2 (FGL2) is an immunoregulatory molecule expressed by macrophages, T cells, and endothelial cells and exists in both membrane-bound and soluble forms (12). Soluble FGL2 has been implicated in immune modulation, inflammatory signaling, and immune vascular interactions and has been shown to be elevated in several chronic inflammatory and autoimmune diseases, including ulcerative colitis, Crohn’s disease, viral hepatitis, liver cirrhosis, hepatocellular carcinoma, and acute pancreatitis (12–15). Reported circulating FGL2 concentrations vary markedly by disease state, with approximate mean serum levels of ∼10 ng/mL in healthy individuals, ∼27–30 ng/mL in inflammatory bowel disease, and up to ∼164 ng/mL in advanced liver cirrhosis, suggesting disease-specific regulation and potential biomarker utility (14, 15). Importantly, despite extensive investigation of downstream cytokines in IBS, FGL2 has not been evaluated in this context, and its relationship with symptom severity or psychological comorbidities remains unknown. Given its upstream immunoregulatory role, FGL2 is a biologically plausible candidate biomarker that may integrate immune activation with the clinical manifestations of IBS-D.

In addition, the established bidirectional communication along the gut-brain axis highlights the relevance of immune-related biomarkers that may reflect both the inflammatory activity and psychological dimensions of IBS (16). In the present study, patient recruitment was based on the Rome III diagnostic criteria to ensure comparability with a substantial body of earlier IBS biomarker and immunological research that employed the same framework (17, 18). The Rome IV criteria define a more stringent and clinically homogeneous IBS population and may better reflect contemporary diagnostic practice. Accordingly, the use of the Rome III criteria may have introduced greater phenotypic heterogeneity, which could influence biomarker–phenotype associations (19, 20). Nevertheless, this approach enabled the recruitment of a well-characterized cohort suitable for exploratory biomarker investigation and allowed our findings to be interpreted in the context of the existing IBS biomarker literature. Future studies should validate serum FGL2 levels in Rome IV-defined IBS populations to further strengthen the clinical applicability and translational relevance of this biomarker.

Therefore, this study aimed to evaluate serum FGL2 levels in patients with IBS-D and examine their associations with disease severity, inflammatory markers, psychological status, and quality of life. Importantly, although immune activation and psychosocial comorbidity are well-recognized features of IBS, FGL2 has not been previously investigated in this disease. Thus, the present work seeks to introduce FGL2 as a novel candidate biomarker that may reflect integrated immune and clinical disease burden rather than establishing a causal role in IBS pathophysiology.

## Materials and methods

### Study population

This case-control study enrolled patients with IBS-D and healthy controls between July 2022 and December 2024 at the Nantong Second People’s Hospital. All participants were aged 18–65 years and provided written informed consent prior to enrollment. The diagnosis of IBS-D was established according to the Rome III criteria by experienced gastroenterologists based on structured clinical interviews and symptom assessment within 6 months prior to enrollment. All patients with IBS-D underwent colonoscopy with histological evaluation to exclude inflammatory bowel disease. Healthy controls were recruited from individuals undergoing routine health examinations at the same hospital during the study period and had no history of chronic gastrointestinal or systemic inflammatory disease. The detailed inclusion and exclusion criteria for patients with IBS-D and healthy controls are summarized in Table 1. The study protocol adhered to the principles of the Declaration of Helsinki. This study was approved by the Ethics Committee of the Nantong Second People’s Hospital (IRB approval number: 2025-060).

**TABLE 1:** Clinical and laboratory characteristics of the study population.

Variables	Controls (*n* = 110)	IBS (*n* = 117)	*P*-value
Age (years)	40.55 ± 9.25	40.87 ± 9.38	0.792
Sex (male, %)	61 (55.5%)	65 (55.6%)	0.988
BMI (kg/m^2^)	22.09 ± 2.72	22.15 ± 2.70	0.867
Smoking	37 (33.6%)	41 (35.0%)	0.824
Education level			0.892
None/primary	23 (20.9%)	24 (20.5%)	
Middle/high school	57 (51.8%)	64 (54.7%)	
University or higher	30 (27.3%)	29 (24.8%)	
WBC (× 10^9^/L)	5.59 ± 1.33	8.15 ± 2.29	< 0.001
Neutrophils (× 10^9^/L)	3.93 ± 1.32	5.17 ± 1.89	< 0.001
TNF-α (pg/mL)	41.60 ± 6.50	57.92 ± 7.91	< 0.001
IL-1β (pg/mL)	9.16 ± 1.75	14.19 ± 2.28	< 0.001
IL-6 (pg/mL)	11.98 ± 1.81	21.09 ± 3.31	< 0.001
IL-17A (pg/mL)	33.87 ± 4.53	48.16 ± 6.16	< 0.001
FGL2 (ng/mL)	86.23 ± 14.89	130.78 ± 25.78	< 0.001

Continuous variables are expressed as mean ± standard deviation, and *t*-test is used to compare the differences between two groups. Categorical variables are expressed in frequency (percentage), and chi square tests are applied to compare the differences between two groups. BMI, body mass index; WBC, white blood cell; TNF-α, tumor necrosis factor- alpha; IL-1β, interleukin-1 beta; IL-6, interleukin-6; IL-17A, interleukin-17A; FGL2, fibrinogen-like protein 2.

### Transcriptomic and volcano plot analysis

Publicly available intestinal mucosal transcriptomic datasets were retrieved from the Gene Expression Omnibus (GEO) database to explore FGL2 expression at the tissue level in patients with IBS. The primary dataset analyzed was GSE36701, which included colonic mucosal biopsy samples from patients with IBS and healthy controls. This dataset comprised 48 IBS samples and 21 control samples, with biopsies obtained from the sigmoid colon, a region commonly implicated in IBS-related mucosal immune alterations. Raw expression data were normalized, and differential expression analysis was performed in R using the *limma* package, which applies linear modeling and empirical Bayes moderation to improve the statistical robustness (21). Genes with a *p*-value < 0.05 and |log2 fold change| > 1 were considered significantly differentially expressed. A volcano plot was generated using the *EnhancedVolcano* package to visualize global transcriptional changes and highlight FGL2 expression in relation to the serum findings (22).

### Clinical data collection

Complete clinical and laboratory data were collected, including age, sex, body mass index (BMI), smoking status, white blood cell (WBC) count, and blood neutrophil counts. Following an overnight fast, 10 mL venous blood samples were collected to assess various biochemical indices, including the WBC and blood neutrophil counts.

### Evaluation of IBS severity

IBS severity was assessed using the IBS severity score system (IBS-SSS) (23, 24). This tool incorporates visual analog scales (VAS) to provide a comprehensive evaluation of symptoms. It combines five key components-pain severity, pain frequency, abdominal distension, dissatisfaction with bowel habits, and interference with daily life-into a single composite score, reflecting the overall symptom burden in IBS. The scale ranges up to 500 points, with higher scores indicating more severe symptoms.

### Evaluation of psychological status

We evaluated participants’ perceived stress over the past month and assessed depressive symptoms using the 9-item Patient Health Questionnaire (PHQ-9) (25). This widely recognized tool provides a detailed assessment of the severity of depression. The PHQ-9 scores were classified into five categories: Minimal (0–4), Mild (5–9), Moderate (10–14), moderately severe (15–19), and Severe (20–27). In our follow-up study, a shift to a lower PHQ-9 category was considered an “improvement in the PHQ-9 score (26). Additionally, we used the Hospital Anxiety and Depression Scale (HADS), a self-administered questionnaire designed to measure anxiety and depression (27). The HADS consists of 14 items-7 assessing anxiety and seven assessing depression-with each item scored from 0 to 3, with higher scores indicating more severe symptoms.

### Assessment of the quality of life

We assessed patients’ quality of life (QOL) using the IBS-QOL questionnaire, which comprises 34 questions (25). This instrument covers eight subscales: food avoidance, dysphoria, body image, interference with activity, health worry, sexual life, social reaction, and relationships. Participants’ responses to all items were summed and converted to a standardized score ranging from 0 to 100, allowing for a comprehensive evaluation of the overall QOL.

### Measurement of serum proteins by ELISA

ELISA was used to measure the serum levels of fibrinogen-like protein 2 (FGL2; Cat#ml063599, Shanghai Enzyme-linked Biotechnology), tumor necrosis factor-alpha (TNF-α; Cat#DTA00D, R&D Systems), interleukin-1 beta (IL-1β; Cat#DY240, R&D Systems), interleukin-6 (IL-6; Cat#D6050B, R&D Systems), and interleukin-17A (IL-17A; Cat#D1700, R&D Systems), and the absorbance was recorded at 450 nm using a microplate reader. The concentrations of TNF-α, IL-1β, IL-6, and IL-17A were determined using a standard curve and expressed as pg/mL.

### Statistical analysis

Statistical analyses were performed using IBM SPSS Statistics version 20.0. Continuous variables with a normal distribution are expressed as mean ± standard deviation, and differences between groups were assessed using Student’s *t*-test or one-way analysis of variance (ANOVA). Categorical variables were compared using the chi-square test. Pearson’s correlation coefficient was used to evaluate the association between serum FGL2 levels and other continuous variables. Receiver operating characteristic (ROC) curve analysis was performed to evaluate the diagnostic performance of serum FGL2 and to determine the optimal cutoff value by maximizing sensitivity and specificity, as previously described (28, 29). A two-sided *p*-value < 0.05 was considered statistically significant.

## Results

### Demographic characteristics

A comprehensive comparative analysis was conducted between individuals diagnosed with IBS and healthy controls. Student’s *t*-test was used to assess differences in clinical and demographic variables. Patients with IBS demonstrated significantly elevated levels of WBC, neutrophils, TNF-α, IL-1β, IL-6, IL-17A, and FGL2 compared to healthy controls. Furthermore, the IBS group was characterized by a lower proportion of individuals with higher educational attainment (university or above) and a greater proportion of individuals with lower levels of education (none/primary and middle/high school). No statistically significant differences were observed between the two groups in terms of age, sex, smoking status, or BMI (*p* > 0.05; Table 2).

**TABLE 2:** Inclusion and exclusion criteria for study participants.

Category	Criteria	Operational definition
Inclusion criteria	Age	18–65 years at enrollment
	IBS-D diagnosis	Diarrhea-predominant IBS diagnosed according to Rome III criteria by an experienced gastroenterologist using structured clinical interview
	Symptom duration	≥6 months prior to enrollment
	Consent	Ability to understand the study and provide written informed consent
Exclusion criteria	Current infection	Fever > 38°C, clinical signs of acute infection, or elevated C-reactive protein (CRP) at screening
	Inflammatory bowel disease	History or current diagnosis of Crohn’s disease or ulcerative colitis, excluded by colonoscopy and histopathology
	Celiac disease	Known diagnosis or exclusion by negative serology and/or biopsy when clinically indicated
	Recent antibiotics or probiotics	Use within 4 weeks prior to enrollment
	Immunomodulatory therapy	Use of systemic corticosteroids, immunosuppressive agents, or biologics within the past 3 months
	Malignancy	Active malignancy or history of malignancy within the past 5 years
	Major psychiatric disorders	Disorders requiring hospitalization (e.g., schizophrenia, bipolar disorder, severe major depressive disorder with hospitalization)
	Recent major surgery	Major gastrointestinal or abdominal surgery within the past 6 months
	Liver dysfunction	ALT or AST > 2 × upper limit of normal
	Renal dysfunction	Estimated glomerular filtration rate < 60mL/min/1.73m^2^
Smoking status	Definition	Classified as current smoker, former smoker, or never smoker; current smokers were not excluded

### FGL2 expression in intestinal tissues

**FIGURE 1:**
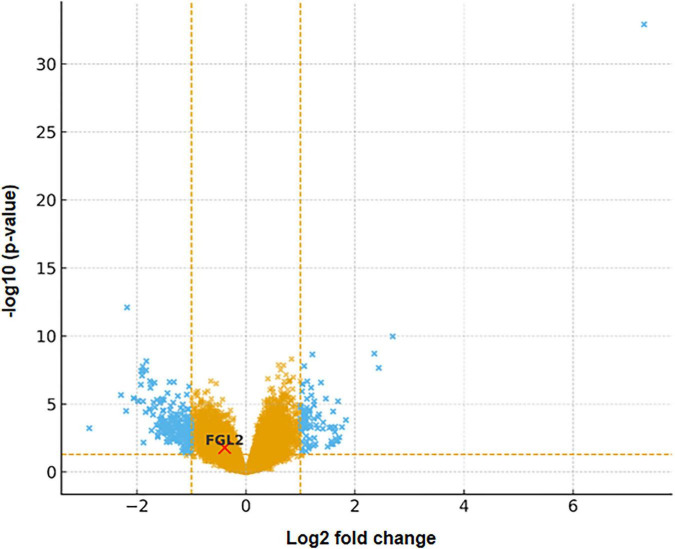
Volcano plot of differential gene expression analysis highlighting FGL2.

Differential expression analysis of colonic mucosal transcriptomic data revealed significant dysregulation of FGL2 mRNA expression in patients with IBS compared to healthy controls (*p* < 0.05) (Figure 1). Although the direction and magnitude of change varied across samples, FGL2 was consistently identified among differentially expressed immune-related genes, indicating altered regulation at the intestinal tissue level in patients with IBS.

### Serum FGL2 levels in patients with IBS

**FIGURE 2:**
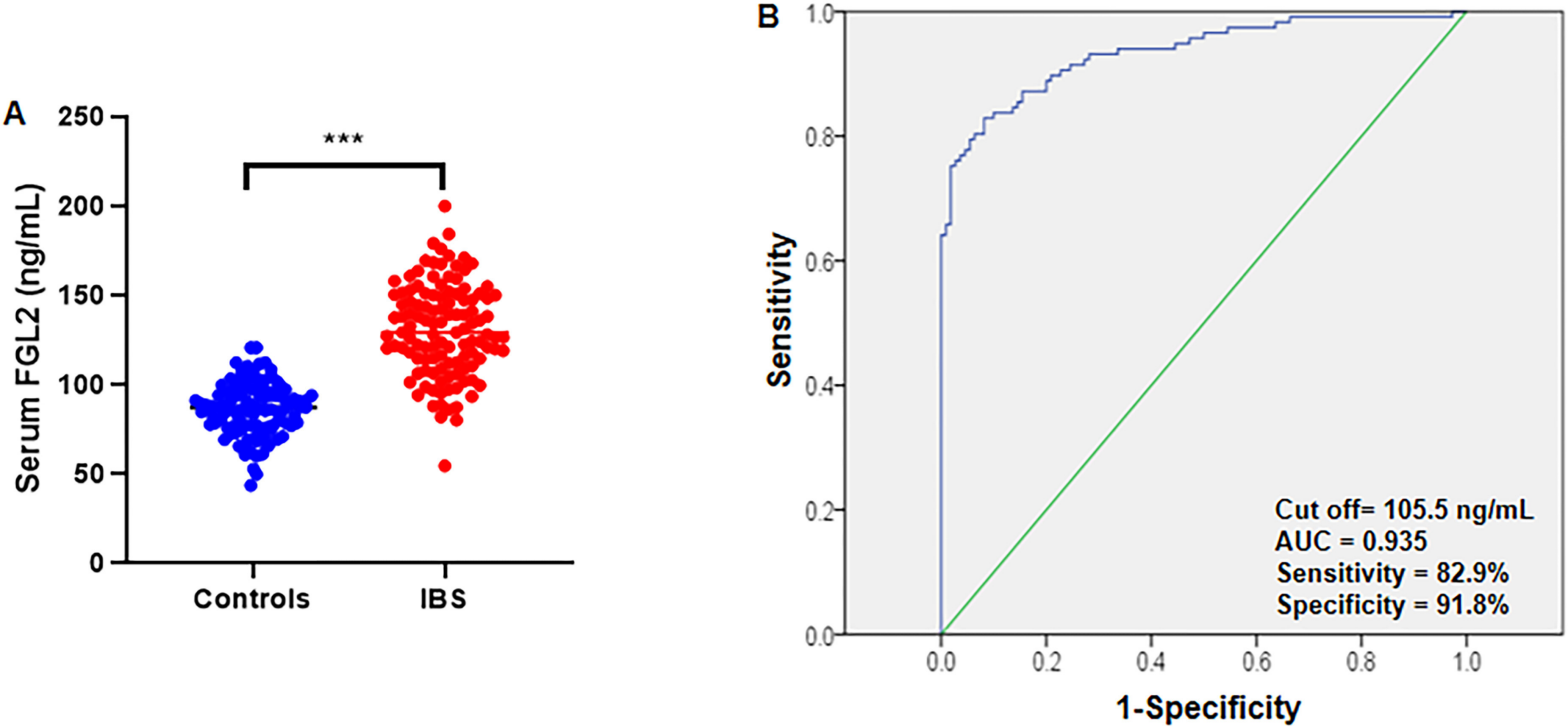
Comparison of serum FGL2 levels between healthy controls and patients with IBS. **(A)** Serum FGL2 levels were higher in IBS patients (*n* = 117) than in control subjects (*n* = 110). Serum FGL2 concentrations were measured by ELISA. **(B)** The ROC curve is used to obtain the critical point of serum FGL2 levels that distinguishes between patients with IBS from healthy controls. The optimal critical point was 105.5 ng/mL. The area under the curve is 0.935. A *t*-test was used to compare the differences between the two groups. ***P < 0.001. IBS, irritable bowel syndrome.

Serum FGL2 levels were significantly higher in patients with IBS than in healthy controls (Figure 2A). The ROC curve analysis demonstrated a strong discriminative performance of serum FGL2 for IBS, with an area under the curve (AUC) of 0.935. The optimal cut-off value was 105.5 ng/mL, yielding a sensitivity of 82.9% and specificity of 91.8% (Figure 2B).

### Correlation between serum FGL2 levels and inflammatory cytokines in patients with IBS

**FIGURE 3:**
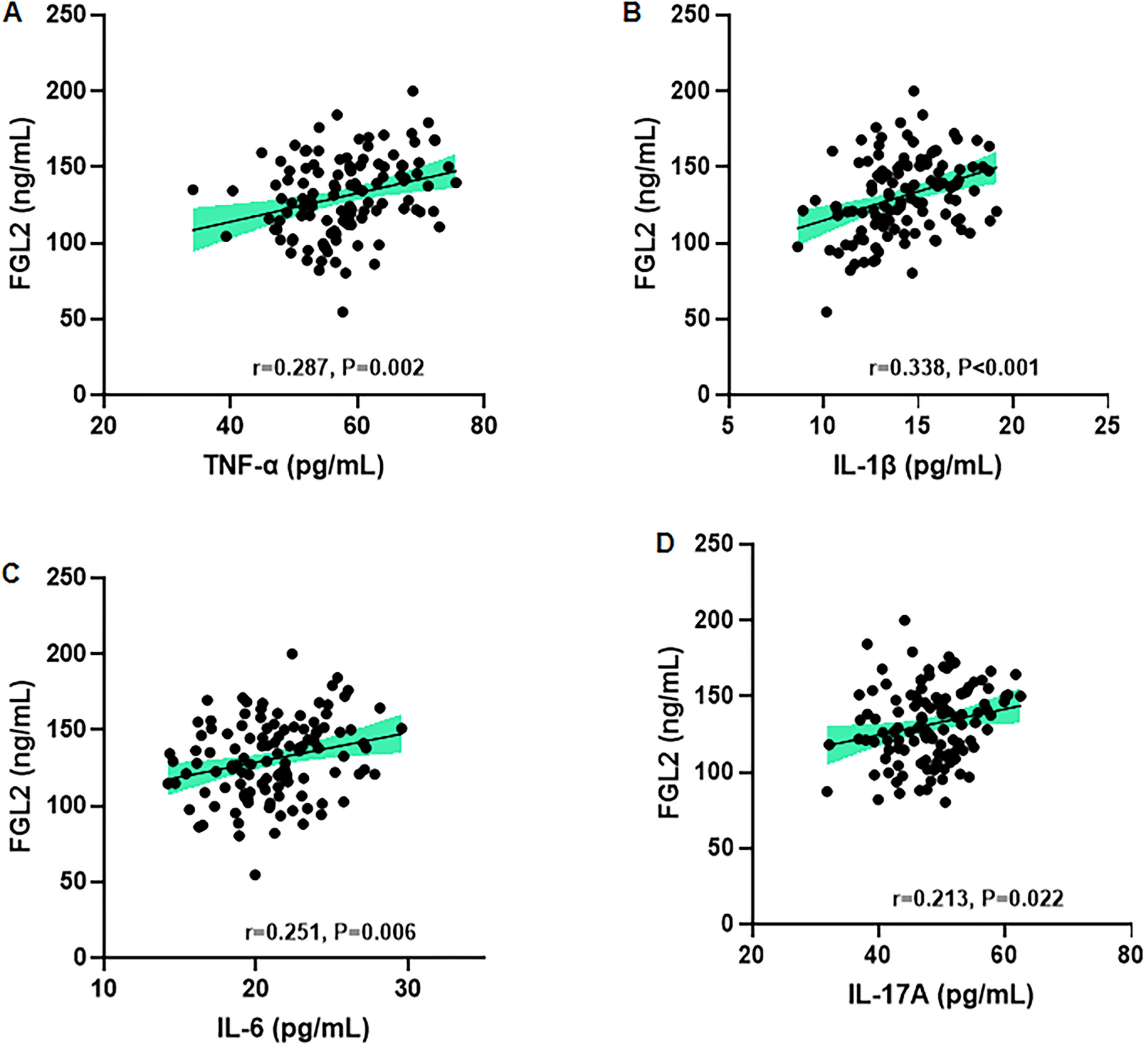
Correlation between serum FGL2 levels and inflammatory cytokines in patients with IBS. Serum FGL2 was positively correlated with **(A)** TNF-α, **(B)** IL-1β, **(C)** IL-6, and **(D)** IL-17A levels. Pearson’s correlation analysis was performed.

Pearson’s correlation analysis revealed significant positive correlations between serum FGL2 levels and pro-inflammatory cytokine levels. Serum FGL2 levels correlated positively with TNF-α (*r* = 0.287, *p* = 0.002), IL-1β (*r* = 0.338, *p* < 0.001), IL-6 (*r* = 0.251, *p* = 0.006), and IL-17A (*r* = 0.213, *p* = 0.022) (Figures 3A–D).

### Correlation between serum FGL levels and disease severity, psychological status in patients with IBS

**FIGURE 4:**
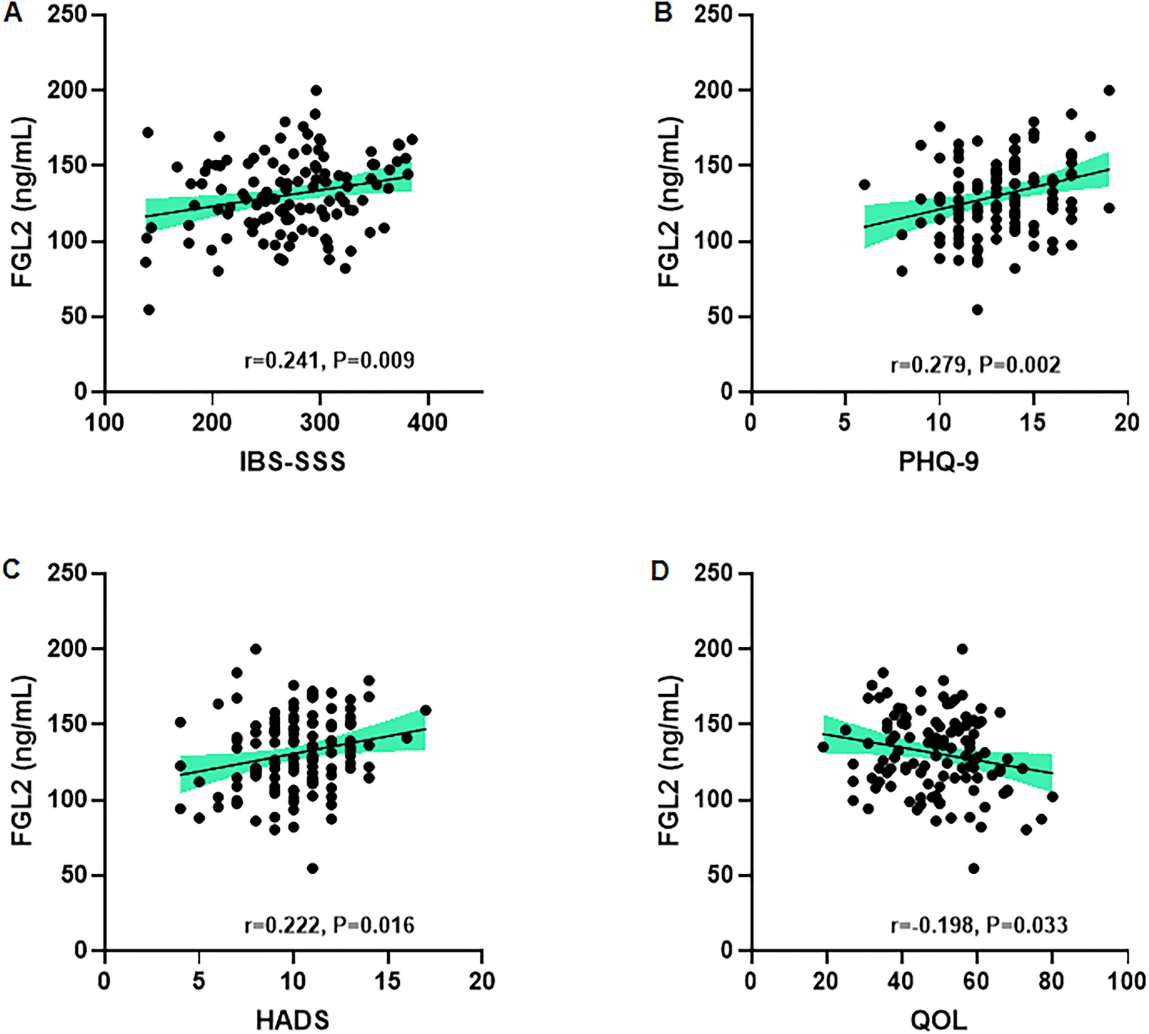
Correlation between serum FGL levels, disease severity, and psychological status in patients with IBS. Serum FGL is positively correlated with **(A)** IBS symptom severity score (IBS-SSS), **(B)** Patient Health Questionnaire-9 (PHQ-9), **(C)** Hospital Anxiety and Depression Scale (HADS), and **(D)** quality of life (QOL) score.

Serum FGL2 levels were positively correlated with IBS symptom severity, as assessed using the IBS Severity Scoring System (IBS-SSS) (*r* = 0.241, *p* = 0.009) (Figure 4A). Significant positive correlations were also observed between serum FGL2 levels and depressive symptoms, as measured by the PHQ-9 (*r* = 0.279, *p* = 0.002) (Figure 4B), as well as anxiety and depression scores, as measured by the HADS (*r* = 0.222, *p* = 0.016) (Figure 4C). In addition, higher serum FGL2 levels were associated with a poorer quality of life, as assessed using the IBS-QOL questionnaire (*r* = 0.198, *p* = 0.033) (Figure 4D).

## Discussion

In the present study, we deliberately focused on patients with IBS-D to reduce clinical heterogeneity and examine a subtype that has been consistently linked to low-grade immune activation and peripheral inflammatory alterations. Accumulating evidence indicates that IBS-D is more frequently associated with elevated circulating pro-inflammatory cytokines and subtle hematological changes than constipation-predominant IBS (IBS-C) or mixed-type IBS (IBS-M) (5, 30). Recent meta-analyses have further demonstrated that biomarkers such as IL-6, TNF-α, and reduced serum albumin are more reliably altered in IBS-D than in other subtypes, whereas findings in IBS-C and IBS-M remain inconsistent, often due to smaller sample sizes and inadequate subtype stratification (8, 31). Therefore, this subtype-specific approach strengthens the internal validity but inevitably limits the generalizability of our findings to the broader IBS population.

Consistent with this rationale, we report for the first time that serum FGL2 levels are significantly elevated in patients with IBS-D, a subtype of IBS characterized by recurrent abdominal pain and loose or frequent stools. In parallel, we confirmed the presence of systemic low-grade inflammation, as reflected by increased WBC and neutrophil counts and elevated circulating pro-inflammatory cytokines, including TNF-α, IL-1β, IL-6, and IL-17A, which have been identified as part of the peripheral immune activation profile in patients with IBS in recent meta-analyses (32). These immune alterations are well aligned with prior IBS literature linking peripheral immune activation to symptom severity, visceral hypersensitivity, and psychological distress within the gut-brain axis framework, as described in comprehensive immunological reviews (33). Importantly, the identification of FGL2 does not propose a novel immune-brain pathway but rather extends the established immune-psychological framework by introducing a previously unexamined immunoregulatory molecule that may integrate the inflammatory and psychosocial disease dimensions.

Our ROC analysis demonstrated that serum FGL2, at a cutoff value of 105.5 ng/mL, distinguished IBS-D patients from healthy controls with high accuracy (AUC = 0.935), supporting its potential utility as a diagnostic biomarker. Moreover, the positive correlations between FGL2 and pro-inflammatory cytokines reinforce the interpretation that elevated FGL2 levels reflect systemic immune activation. The additional associations with IBS-SSS, PHQ-9, HADS, and reduced quality of life further suggest that FGL2 elevations may mirror both physiological inflammation and the psychosocial burden of disease, which are tightly intertwined in patients with IBS.

FGL2 is a multifunctional immune regulator with context-dependent effects on immune responses. The soluble form of FGL2 (sFGL2), predominantly secreted by regulatory T cells, exerts immunosuppressive effects by inhibiting dendritic cell maturation and T cell activation. In contrast, the membrane-bound form (mFGL2), expressed on macrophages and endothelial cells, functions as a prothrombinase, promoting fibrin deposition and immune vascular interactions (34, 35). Experimental models of intestinal inflammation have shown that FGL2 deficiency exacerbates colitis and enhances M1 macrophage polarization, underscoring its role in maintaining the mucosal immune balance (36). Within the context of IBS, which is now increasingly recognized as a disorder involving low-grade mucosal immune activation, epithelial barrier dysfunction, and altered gut-brain signaling (37), altered FGL2 expression may reflect dysregulation within the intestinal immune coagulation microenvironment.

Based on our circulating data and reanalysis of mucosal transcriptomic datasets, we cautiously speculate that IBS-D may involve a relative imbalance between FGL2 isoforms, characterized by reduced mucosal mFGL2 expression, alongside compensatory secretion of sFGL2 into the circulation. This shift may represent an attempted immunoregulatory response to dampen local inflammation while simultaneously manifesting as elevated systemic FGL2 levels. However, given the cross-sectional design and the inability of bulk transcriptomic data to distinguish FGL2 isoforms, this interpretation remains hypothetical and warrants validation through isoform-specific and spatially resolved analysis.

It is also important to contextualize our findings within the broader IBS literature, which demonstrates substantial heterogeneity in systemic inflammatory profiles. Not all IBS cohorts exhibit measurable peripheral inflammation, particularly when unselected or mixed-subtype populations are examined, suggesting that immune activation in IBS is context- and subtype-dependent rather than universal (5, 38). Proteomic studies have further indicated that although inflammatory signatures can reliably distinguish IBS from inflammatory bowel disease, these profiles do not necessarily reflect overt systemic inflammation in IBS *per se*, underscoring the spectrum-based nature of immune involvement in functional gastrointestinal disorders (39, 40). Meta-analyses and systematic reviews consistently report that while certain circulating cytokines are statistically elevated at the group level, effect sizes are modest, and inter-study heterogeneity remains high, likely reflecting variability in diagnostic criteria, patient selection, sampling strategies, and assay methodologies (41, 42). Collectively, these observations underscore the need for caution when extrapolating immunological findings beyond carefully phenotyped subgroups, such as IBS-D, in which inflammatory alterations appear more consistently detectable (4, 43).

Our absolute serum FGL2 concentrations were higher than those previously reported for some inflammatory diseases, likely due to variability among enzyme-linked immunosorbent assay (ELISA) platforms, including antibody specificity, calibration standards, and whether total or isoform-specific FGL2 is measured. In acute pancreatitis, serum sFGL2 > 200ng/mL predicted delirium using different assay systems (34, 44). Similar variability has been observed in inflammatory bowel disease (IBD) studies, highlighting the need for cautious interpretation of absolute values. Nonetheless, the consistent relative elevation of FGL2 in IBS-D versus controls supports its biological and diagnostic relevance (12).

Finally, the observed associations between FGL2 levels and depressive symptoms, anxiety, and impaired quality of life should not be interpreted as evidence of a direct neuroimmune mechanism. FGL2 has not been shown to cross the blood-brain barrier or act centrally in humans. Rather, these relationships likely reflect shared systemic immune-inflammatory pathways that have been repeatedly implicated in the psychosocial dimensions of IBS (45). Thus, FGL2 may serve as a peripheral biomarker of immune dysregulation linked to both symptom severity and psychological burden rather than a direct mediator of brain function.

We also noted lower educational attainment among patients with IBS than among healthy controls. Education may act as a proxy for socioeconomic stressors, health literacy, and chronic life stress exposure, all of which can influence IBS vulnerability and symptom perception. Importantly, no differences were observed in age, sex, smoking status, or body mass index, supporting the robustness of our immunological findings, independent of these common confounders. Taken together, our findings suggest that while low-grade immune activation and elevated FGL2 levels, in particular, may be more prominent in IBS-D, systemic inflammation is not a universal feature of all IBS populations. These results highlight the importance of subtype-specific investigations and support FGL2 as a promising integrative biomarker linking immune activation with clinical and psychosocial disease burden in IBS-D. Future longitudinal and mechanistic studies across IBS subtypes are needed to clarify the temporal dynamics, tissue specificity, and functional significance of FGL2 in functional gastrointestinal disorders.

## Limitations

Our study had several limitations. First, it was conducted at a single center and employed a cross-sectional design, which may limit causal inference and reduce generalizability to broader populations. Additionally, only patients with IBS-D were included, and other subtypes, such as IBS-C and IBS-M were not examined. Future studies should include multiple IBS subtypes to determine whether the associations between serum FGL2 levels and clinical features are consistent across presentations. Second, the diagnosis of IBS-D was based on the Rome III criteria rather than the more recently established Rome IV criteria, which may have resulted in a more heterogeneous study population and could potentially influence the observed associations between serum FGL2 levels and clinical or psychological phenotypes. Third, our study did not include external or split-sample validation of the diagnostic performance of serum FGL2 levels, which may affect the generalizability of the diagnostic findings. Validation in independent cohorts is warranted to confirm and strengthen the diagnostic utility. Fourth, transcriptomic analysis was based on publicly available bulk RNA datasets derived from colonic mucosal biopsies. These data do not allow for cell type-specific or isoform-specific resolution of FGL2 expression. Therefore, the transcriptomic findings should be interpreted as exploratory and supportive rather than definitive evidence of the mechanism. Finally, mucosal biopsy or immunohistochemistry was not performed to assess tissue-level FGL2 expression. Future studies incorporating tissue analysis and mechanistic investigations into how FGL2 interacts with gut mucosal immunity and the neuro-immune axis are needed to clarify its role in IBS pathophysiology.

## Conclusion

This study provides the first evidence that serum FGL2 levels are elevated in patients with IBS-D and correlate with inflammatory cytokines, symptom severity, psychological distress, and reduced quality of life. Transcriptomic analyses indicate the differential expression of FGL2 isoforms in intestinal tissue, with reduced membrane-bound FGL2 potentially contributing to local microinflammation, whereas secreted FGL2 accumulates in the serum. These results suggest that FGL2 may link the inflammatory and psychosocial dimensions of IBS and represents a novel candidate biomarker. While the immune-psychological associations largely confirm prior observations, the isoform imbalance and serum elevation of FGL2 are unique contributions. Causality cannot be inferred given the cross-sectional design. Further studies in larger, independent cohorts and across IBS subtypes are warranted to validate these findings and clarify the mechanistic role of FGL2 in IBS.

## Data Availability

The datasets presented in this study can be found in online repositories. The names of the repository/repositories and accession number(s) can be found in the article/supplementary material.
